# Supplementary material for: Psychosocial functioning of adolescents with ADHD in the family, school and peer group: A scoping review protocol
Source: PLoS One. 2022 Jun 17;17(6):e0269495. doi: 10.1371/journal.pone.0269495 (PMC9205482; doi:10.1371/journal.pone.0269495)
Supplement: S1 Appendix — (PDF) [file pone.0269495.s001.pdf]

## **S1 Appendix.** The exemplary search strings for a preliminary search.

### The search string for the Cochrane Database of Systematic Reviews:

(ADHD OR ADD OR "AD HD" OR ADHS OR "attention deficit" OR hyperactive* OR hyperkine* OR inattention OR impulsivity):ti,ab,kw AND (adolescen* OR teen* OR youth* OR pube* OR young* OR juvenile OR minor* OR pupil* OR student*):ti,ab,kw AND ("systematic review*" OR "scoping review*" OR scope OR metaanaly* OR “meta-analy*” OR "meta analy*"):ti,ab,kw

### The search string for the PubMed:

((ADHD[Title/Abstract] OR ADD[Title/Abstract] OR "AD HD"[Title/Abstract] OR ADHS[Title/Abstract] OR "attention deficit"[Title/Abstract] OR hyperactive*[Title/Abstract] OR hyperkine*[Title/Abstract] OR inattention[Title/Abstract] OR impulsivity[Title/Abstract]) AND (adolescen*[Title/Abstract] OR teen*[Title/Abstract] OR youth*[Title/Abstract] OR pube*[Title/Abstract] OR young*[Title/Abstract] OR juvenile[Title/Abstract] OR minor*[Title/Abstract] OR pupil*[Title/Abstract] OR student*[Title/Abstract])) AND ("systematic review*"[Title/Abstract] OR "scoping review*"[Title/Abstract] OR scope[Title/Abstract] OR metaanaly*[Title/Abstract] OR "meta-analy*"[Title/Abstract] OR "meta analy*"[Title/Abstract])
