# Supplementary material for: Psychosocial functioning of adolescents with ADHD in the family, school and peer group: A scoping review protocol
Source: PLoS One. 2022 Jun 17;17(6):e0269495. doi: 10.1371/journal.pone.0269495 (PMC9205482; doi:10.1371/journal.pone.0269495)
Supplement: S3 Appendix — (PDF) [file pone.0269495.s003.pdf]

S3 Appendix. Search strings for Scopus and EBSCO databases.

SCOPUS:

(TITLE-ABS-KEY ((adhd OR {AD HD} OR {AD/HD} OR adhs OR "Attention Deficit Hyperactivity Disorder" OR "Attention Deficit and Hyperactivity Disorder" OR "Attention Deficit Disorder" OR "Hyperkinetic Disorder" OR hkd OR "Hyperkinetic syndrome") AND ((adolescen*) OR (teen*) OR (youth*) OR (pube*) OR (young*) OR juvenile OR (minor*) OR (pupil*) OR (student*)) AND ("social function*" OR "psychosocial function*" OR "psycho social function*" OR "famil* functioning" OR "functioning in famil*" OR "school functioning" OR "functioning in school" OR "functioning at school" OR "academic* functioning" OR "peer group functioning" OR "peer functioning" OR "functioning in peer group*")) LANGUAGE (english) DOCTYPE (ar) PUBYEAR > 1986)

EBSCO[1]:

("ADHD" OR "AD HD" OR "AD/HD" OR ADHS OR "Attention Deficit Hyperactivity Disorder" OR "Attention?Deficit Hyperactivity Disorder" OR "Attention?Deficit?Hyperactivity Disorder" OR "Attention Deficit and Hyperactivity Disorder" OR "Attention Deficit Disorder" OR "Hyperkinetic Disorder" OR "HKD" OR "Hyperkinetic syndrome") AND (adolescen* OR teen* OR youth* OR pube* OR young* OR juvenile OR minor* OR pupil* OR student*) AND ("social function*" OR "psychosocial function*" OR "psycho-social function*" OR "famil* functioning" OR "functioning in famil*" OR "school functioning" OR "functioning in school" OR "functioning at school" OR "academic* functioning" OR "peer?group functioning" OR "peer functioning" OR "functioning in peer group*")

Limiters[2] - Scholarly (Peer Reviewed) Journals; Published Date: 19870101-; Language: English

[1] Databases under EBSCO: Academic Search Ultimate, ERIC, MEDLINE.

[2] The default search in EBSCO databases searches several fields, including the Article Title, the Abstract and Keywords, so they are not added as limiters.
