# Supplementary material for: Psychosocial functioning of adolescents with ADHD in the family, school and peer group: A scoping review protocol
Source: PLoS One. 2022 Jun 17;17(6):e0269495. doi: 10.1371/journal.pone.0269495 (PMC9205482; doi:10.1371/journal.pone.0269495)
Supplement: S4 Appendix — (PDF) [file pone.0269495.s004.pdf]

S4 Appendix. Data extraction instrument (preliminary table).

No.	Author(s)	Year of publication	Country of origin	Aim(s)	Population	Sex	Age details (M, SD, %, etc.)	Sample size (in general and divided by groups)	Formal diagnosis (YES / NO)	Basis of diagnosis (DSM, ICD, others). Who rated?
Study 1										
Study 2										
Study 3										

(continuation)

Comorbidities (YES / NO) What exactly?	Components of psychosocial functioning or its domains (Q1)	Method(s) / study design / type of study	Tools / questionnaires used	Who was surved? (adolescents, parents, teachers, etc.)	Details of intervention (if applicable)	Comparator(s) (if applicable)

(continuation)

Method(s) of analysis	Outcomes	Key findings for Q2 (overall psychosocial functioning)	Key findings for Q3 (functioning in family)	Key findings for Q4 (functioning at school)	Key findings for Q5 (peer functioning)
