# Supplementary material for: Psychosocial functioning of adolescents with ADHD in the family, school and peer group: A scoping review protocol
Source: PLoS One. 2022 Jun 17;17(6):e0269495. doi: 10.1371/journal.pone.0269495 (PMC9205482; doi:10.1371/journal.pone.0269495)
Supplement: S5 Appendix — (PDF) [file pone.0269495.s005.pdf]

S5 Appendix. Draft tabular presentation of the PCC components.

PARAMETER	RESULTS (number)
**Publications on psychosocial functioning concept**	
On overall psychosocial functioning	
On functioning in family	
On functioning at school	
On peer functioning	
**Types of quantitative studies**	
Randomized controlled trials	
Non-randomized controlled trials	
Quasi-experimental studies	
Before-and-after studies	
Others	
**Types of qualitative studies**	
Grounded theory	
Ethnography	
Phenomenology	
Action research	
Others	
**Population – sample size**	
**% male**	
**Adolescence stage**	
Early adolescence	
Middle adolescence	
Late adolescence	
**Subtypes (presentations) of ADHD**	
Predominantly Inattentive	
Predominantly Hyperactive-Impulsive	
Combined	
Not defined	
**Subgroups with comorbidities***	
**Subgroups with pharmacological treatment***	
**Countries***	

* Categories unknown at the protocol stage
