# Supplementary material for: Psychosocial functioning of adolescents with ADHD in the family, school and peer group: A scoping review protocol
Source: PLoS One. 2022 Jun 17;17(6):e0269495. doi: 10.1371/journal.pone.0269495 (PMC9205482; doi:10.1371/journal.pone.0269495)
Supplement: S6 Appendix — (PDF) [file pone.0269495.s006.pdf]

**S6 Appendix.** Components of psychosocial functioning in evidence sources (draft table).

| | OVERALL SOCIAL FUNCTIONING | | | | | FUNCTIONING IN THE FAMILY | | | | FUNCTIONING AT SCHOOL | | | | | PEER FUNCTIONING | | | |
|---|---|---|---|---|---|---|---|---|---|---|---|---|---|---|---|---|---|---|
| Reference | social skill | social network | alienation | social support | interp. connectedness | family relationships | family dysfunction | family communication | child-parent relation | motivation to learn | school outcomes | school attendance | student-teacher relation | academic impairment | peer relationships | peer rejection | peer status | peer victimization |
| Author 1 (YEAR) | | | | x | | | | | x | | | | | | | | x | |
| Author 2 (YEAR) | x | | | | | | | | | x | x | x | | | | | | |
| Author 3 (YEAR) | | | | | | | | | | | | | | | x | | x | x |
