# Supplementary material for: Psychosocial functioning of adolescents with ADHD in the family, school and peer group: A scoping review protocol
Source: PLoS One. 2022 Jun 17;17(6):e0269495. doi: 10.1371/journal.pone.0269495 (PMC9205482; doi:10.1371/journal.pone.0269495)
Supplement: Attachment — Submitted filename: PLOS scoping review comments.docx [file pone.0269495.s007.docx]

1. **Summary of the research and your overall impression**

The authors describe a planned scoping review that explores an important area in the field of adolescent mental health and wellbeing. They clearly outline the methodological steps that will be undertaken to identify and synthesise studies that discuss the topic of psychosocial difficulties among adolescents with ADHD. Bringing together the literature on 3 key areas of adolescents’ life (i.e. family, school and peer group) would be a valuable contribution to knowledge that could inform future research. However, there are a few minor issues that could be addressed at this proposal stage which could benefit the study process and outcomes.

2. **Minor issues to be considered**

- The authors stated that other/similar review have been conducted “relatively long ago”. Would it be possible for the authors to explicitly state a timeframe? For example, “more than 5 years ago”.
- The authors used “etc” a number of times in the manuscript. Can the authors rephrase these sentences to avoid using “etc” as this is less common for academic submissions?
- The authors describe the propose study as innovative. However, the argument that “ADHD is still being researched and we still know relatively little about it in youth” is a bit unclear. The authors may wish to further develop this point possibly narrowing it to focus on psychosocial difficulties in adolescents. This is important as there is a wealth of evidence on ADHD and youth as it relates to other fields like medicine and nutrition.
- The authors may wish to provide a citation to support their justification for excluding information not published in peer reviewed journals. This is important as one of the advantages of the scoping review methodology is that is gives researchers the opportunities to search more broadly using grey literature sources to identify information that is usually missed in traditional systematic reviews. It is possible that grey literature searches were conducted in the preliminary searches as the authors mentioned Figshare and OSF.
- The authors described piloting 10% of the studies during screening and data extraction before independently working on the remaining 90%. However, it is not clear if any verification or cross-checking or comparisons will be done to ensure further accuracy, reliability and consistency. Although not explicitly stated in the JBI guidance would the authors consider this as a limitation?
- Similarly, the authors may wish to acknowledge that not including the “optional consultation phase” proposed by other scoping review methodologist (Arksey and O’Malley framework) could be a limitation or a consideration for future research? Owing to the research questions proposed there might be an opportunity here for valuable input from lived experience experts.
